# Roles of Category, Shape, and Spatial Frequency in Shaping Animal and Tool Selectivity in the Occipitotemporal Cortex

**DOI:** 10.1523/JNEUROSCI.3064-19.2020

**Published:** 2020-07-15

**Authors:** Chenxi He, Shao-Chin Hung, Olivia S. Cheung

**Affiliations:** Department of Psychology, Division of Science, New York University Abu Dhabi, Abu Dhabi, United Arab Emirates

**Keywords:** animacy, category, fMRI, gist statistics, multivoxel pattern analysis, ventral visual pathway

## Abstract

Does the nature of representation in the category-selective regions in the occipitotemporal cortex reflect visual or conceptual properties? Previous research showed that natural variability in visual features across categories, quantified by image gist statistics, is highly correlated with the different neural responses observed in the occipitotemporal cortex. Using fMRI, we examined whether category selectivity for animals and tools would remain, when image gist statistics were comparable across categories. Critically, we investigated how category, shape, and spatial frequency may contribute to the category selectivity in the animal- and tool-selective regions. Female and male human observers viewed low- or high-passed images of round or elongated animals and tools that shared comparable gist statistics in the main experiment, and animal and tool images of naturally varied gist statistics in a separate localizer. Univariate analysis revealed robust category-selective responses for images with comparable gist statistics across categories. Successful classification for category (animals/tools), shape (round/elongated), and spatial frequency (low/high) was also observed, with highest classification accuracy for category. Representational similarity analyses further revealed that the activation patterns in the animal-selective regions were most correlated with a model that represents only animal information, whereas the activation patterns in the tool-selective regions were most correlated with a model that represents only tool information, suggesting that these regions selectively represent information of only animals or tools. Together, in addition to visual features, the distinction between animal and tool representations in the occipitotemporal cortex is likely shaped by higher-level conceptual influences such as categorization or interpretation of visual inputs.

**SIGNIFICANCE STATEMENT** Since different categories often vary systematically in both visual and conceptual features, it remains unclear what kinds of information determine category-selective responses in the occipitotemporal cortex. To minimize the influences of low- and mid-level visual features, here we used a diverse image set of animals and tools that shared comparable gist statistics. We manipulated category (animals/tools), shape (round/elongated), and spatial frequency (low/high), and found that the representational content of the animal- and tool-selective regions is primarily determined by their preferred categories only, regardless of shape or spatial frequency. Our results show that category-selective responses in the occipitotemporal cortex are influenced by higher-level processing such as categorization or interpretation of visual inputs, and highlight the specificity in these category-selective regions.

## Introduction

As the human brain transforms visual inputs into conceptual representations, the occipitotemporal cortex is a potential locus where such transformation may occur (Carlson et al., 2014; Clarke and Tyler, 2014, 2015). While category-selective regions for animals and tools in the occipitotemporal cortex have been widely documented (Chao et al., 1999; Downing et al., 2006; Gerlach, 2007; Martin, 2007; Kriegeskorte et al., 2008; Mahon and Caramazza, 2009; Mur et al., 2012; Proklova et al., 2016), the nature of the representations in these regions remains controversial. It remains unresolved regarding the following: (1) the extent that these regions represent visual or conceptual information, as visual and conceptual differences among categories often covary (Rice et al., 2014; Watson et al., 2014); and (2) the extent that these regions represent information content of the preferred categories only, or multiple categories (Kanwisher, 2010; Haxby et al., 2001). Here we disentangled the influences of category, shape, and spatial frequency in shaping the neural representation in the animal- and tool-selective regions in the occipitotemporal cortex.

The lateral and medial parts of the occipitotemporal cortex are selectively activated when viewing images of animals and tools, respectively. Although several studies suggested that the differential neural responses may be due to conceptual differences among categories (Chao et al., 1999; Kriegeskorte et al., 2008), items from different categories often comprise systematic visual differences. For instance, animals generally have curvy shapes, while tools tend to be elongated (Almeida et al., 2014; Chen et al., 2018). Curvilinearity and rectilinearity appear to be sufficient for distinguishing between images of animals and manmade objects, even when the images were rendered unrecognizable (Long et al., 2017, 2018; Zachariou et al., 2018). Comparison of the lateral versus the medial occipitotemporal cortex shows sensitivity not only to animals versus tools, but also to curvilinear versus rectilinear shapes (Op de Beeck et al., 2008; Srihasam et al., 2014), and low spatial frequency (LSF) versus high spatial frequency (HSF; Rajimehr et al., 2011; Mahon et al., 2013; Canário et al., 2016; but see Berman et al., 2017). Moreover, differences in image gist statistics across categories can account for category-selective response patterns in the occipitotemporal cortex (Rice et al., 2014; Watson et al., 2014; Coggan et al., 2016, 2019). Therefore, it is often difficult to tease apart the extent that visual or conceptual features associated with a category contribute to category-selective responses.

Several recent studies have suggested that the occipitotemporal cortex is sensitive to both visual and category information, following a posterior-to-anterior axis (Bracci and Op de Beeck, 2016; Kaiser et al., 2016; Proklova et al., 2016). However, the relative influences of visual and category information on the neural representations remain unclear, especially within the regions that are most responsive to a particular category. Specifically, animal-selective regions may be sensitive to a combination of animal features, round shapes, and LSF, whereas tool-selective regions may be sensitive to tool features, elongated shapes, and HSF. Alternatively, representations in the category-selective regions may primarily be driven by only a certain type of visual or conceptual information, such as animal or tool features that are independent of shapes or spatial frequencies.

We used a diverse image set of animals and tools that shared comparable gist statistics across categories to examine the effects of Category (animals vs tools), Shape (round vs elongated), and SF (LSF vs HSF) in the category-selective regions for animals and tools. We first used univariate analysis to examine the response magnitudes in these regions among the categories, shapes, and spatial frequencies. We then used support vector machine (SVM) classification to further examine the degree that category, shape, and spatial frequency information is represented in the neural response patterns in these regions. We also used representational similarity analysis (RSA) to compare the neural response patterns in these regions to the theoretical models that represent specialized versus distributed information content for categories, shapes, and spatial frequencies (i.e., Animal/Tool/Round/Elongated/LSF/HSF models vs Category/Shape/SF models).

## Materials and Methods

### 

#### Participants

Twenty healthy, right-handed adults (13 female, 7 male), between 19 and 35 years of age (mean age, 23.9; SD, 4.2), from the New York University community took part in the main study. All participants had normal or corrected-to-normal vision. All participants provided informed consent and were compensated for their participation.

#### Materials

**Figure 1.**
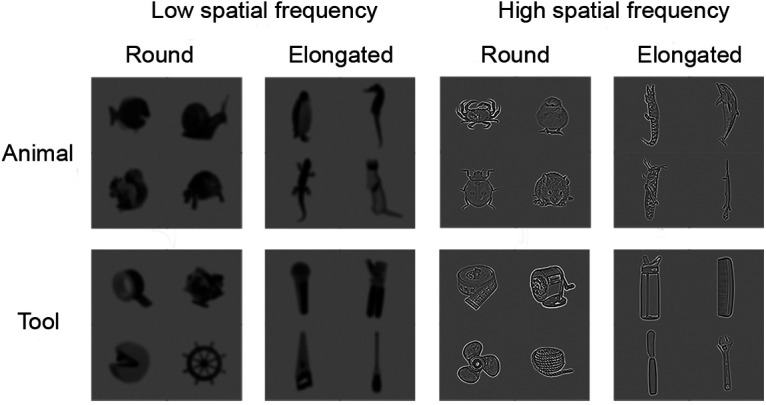
Sample stimuli in each of the Category (animals vs tools), Shape (round vs elongated), and Spatial Frequency (low vs high) conditions. The contrast of HSF images here was increased for illustration purpose, whereas the contrast of the HSF images used in the experiment was decreased to equate visibility between LSF and HSF images. A total of 16 animals and 16 tools were used, each with 16 exemplar images.

A total of 512 grayscale images were used in the main study, including 16 animals and 16 tools, with 16 exemplars for each item. An exemplar of each of the animal and tool stimuli were illustrated in Figure 1. The complete stimulus set can be found on https://osf.io/62jyh/. Half of the animal and tool items were of round shape, and the other half were of elongated shape. We selected these animals and tools primarily to minimize differences in image statistics between the two categories for each shape, and to maximize the representations for each category. The animals were from a wide range of subcategories including mammals, birds, fish, reptiles, and insects. While several elongated tools were typically associated with specific single-hand manipulations (e.g., comb, screwdriver), the round tools included a variety of manipulable objects (e.g., pencil sharpener, rope), and tools that might require manipulations with both hands or electric power (e.g., steering wheel, circular saw, propeller). While we attempted to minimize any systematic, nonvisual differences that might be confounded with the shape manipulation, it was impossible to entirely rule out this possibility. All images were 5.6° of visual angle.

All images were spatially filtered with either low- or high-pass filters (≤1.6 or 8–9.6 cycles/° for LSF and HSF conditions) respectively. The LSF and HSF ranges were selected for comparable visibility between the stimuli in a behavioral pilot study. The main concern for equating visibility between LSF and HSF was both due to the fact that visibility alone could modulate the overall responses in the occipitotemporal cortex (Ludwig et al., 2016) and to the previous findings that HSF images are often more visible than LSF images (de Gardelle and Kouider, 2010). Therefore, we attempted to minimize such a potential confound of differential visibility between LSF and HSF by adjusting the contrast of the stimuli for comparable visibility between LSF and HSF images (Cheung and Bar, 2014). In the pilot study, participants (*N* = 11) viewed contrast-adjusted LSF and HSF stimuli that were briefly presented (300 ms), and judged whether the real-world size of the item was larger or smaller than a typical shoebox. We found comparable performance between the contrast-adjusted LSF and HSF images. While there was a speed–accuracy trade-off between LSF and HSF images [mean response time (RT): LSF, 648.8 ± 121.4 ms; HSF, 672.7 ± 117 ms; *F*_(1,10)_ = 17.6, *p* = 0.002; mean accuracy: LSF, 0.77 ± 0.08; HSF, 0.79 ± 0.08; *F*_(1,10)_ = 12.3, *p* = 0.006], the inverse efficiency that integrated accuracy and RT measures showed no statistical difference in performance for LSF and HSF images (*F*_(1,10)_ = 0.4, *p* = 0.53).

**Figure 2.**
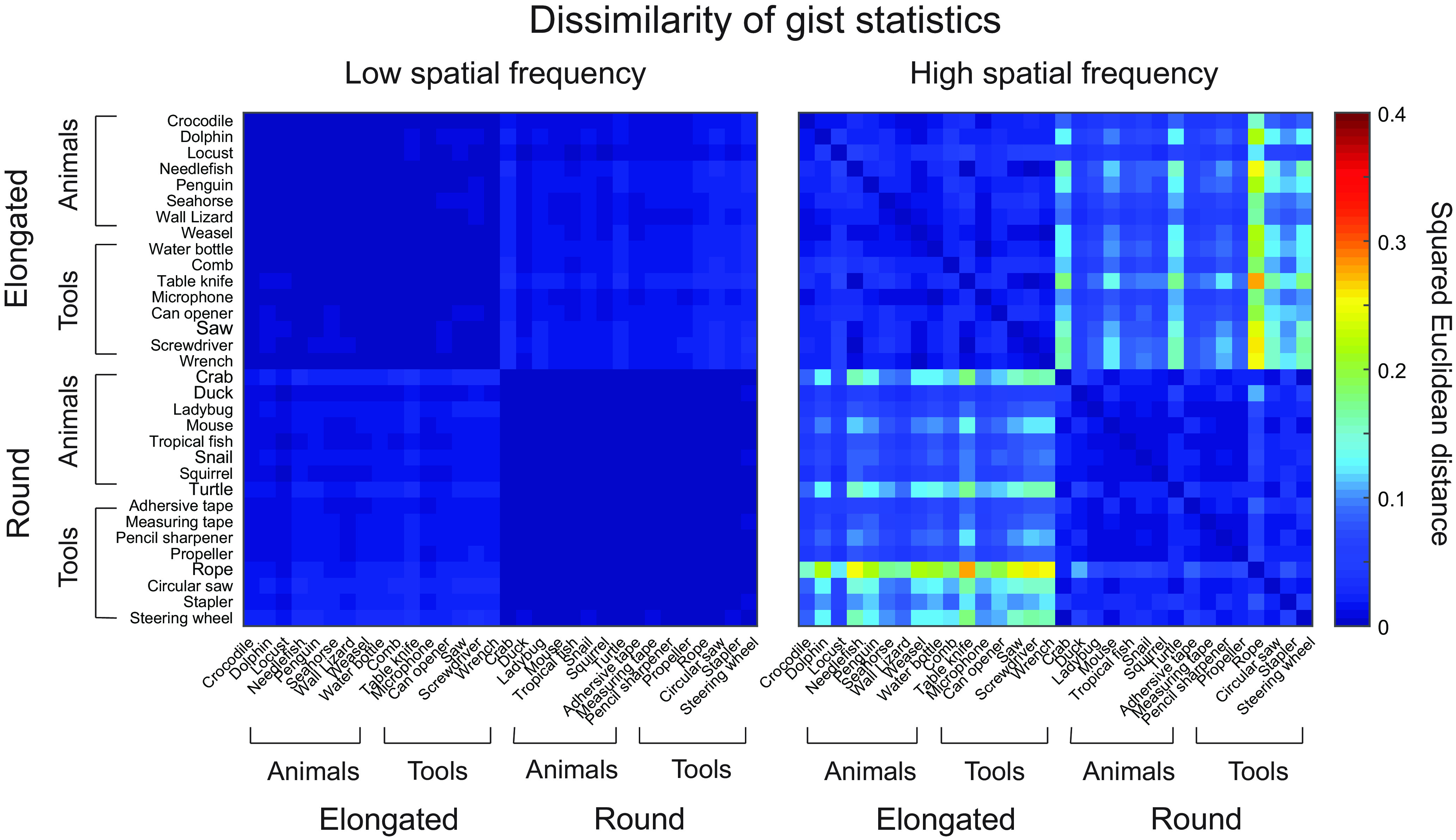
Pairwise dissimilarity (squared Euclidean distance) of gist statistics of low and high spatial frequency images showed significant differences between elongated and round shapes in each category, but no significant differences between animals and tools for each shape.

More importantly, to quantitatively measure the holistic shape properties of the images across categories, a gist descriptor was calculated for each image (Oliva and Torralba, 2001; see also Rice et al., 2014). Specifically, a series of Gabor filters across eight orientations and four spatial frequencies was applied to the images. Each of the resulting 32 filtered images was then segmented into a 4 × 4 grid, and the energy was averaged within each grid to produce final gist statistics containing 512 values. We then calculated the pairwise dissimilarities of the gist statistics across items within and across shapes or categories, by squared Euclidean distance (Fig. 2; Oliva and Torralba, 2001; He and Cheung, 2019). As expected, round and elongated shapes showed significant differences in gist statistics: the average within-shape dissimilarity was significantly lower than the average cross-shape dissimilarity for images across categories and spatial frequencies (e.g., a squirrel and a turtle in LSF vs a squirrel and a penguin in LSF; LSF animal: *t*_(118)_ = −15.2, *p* < 0.0001; LSF tool: *t*_(118)_ = −22.7, *p* < 0.0001; HSF animal: *t*_(118)_ = −11.4, *p* < 0.0001; HSF tool: *t*_(118)_ = −12.2, *p* < 0.0001). Critically, however, the average within-category dissimilarity was comparable to the average cross-category dissimilarity for images across different shapes and spatial frequencies (e.g., a squirrel and a turtle in LSF vs a squirrel and a steering wheel in LSF; LSF round: *t*_(118)_ = −0.7, *p* = 0.47; LSF elongated: *t*_(118)_ = −1.3, *p* = 0.21; HSF round: *t*_(118)_ = −0.1, *p* = 0.92; HSF elongated; *t*_(118)_ = −1.4, *p* = 0.16), suggesting no systematic difference in gist statistics between the animal and tool images.

The localizer used full-spectrum, grayscale images of 60 animals and 60 tools with natural shape variations (He et al., 2013; Konkle and Caramazza, 2013), and the scrambled versions of such images. Each image was 5° of visual angle. The animals included various mammals, birds, fish, reptiles, and insects. The tools included items used in various environments such as homes, offices, hospitals, construction sites, and fields.

#### Experimental design and statistical analysis

##### Design and procedure of the main fMRI experiment.

In the main experiment, half of the 16 animals and 16 tools were randomly allocated into the LSF or HSF conditions for each participant. Each participant saw a total of 8 animals and 8 tools (of 16) in the LSF condition (4 round and 4 elongated animals, 4 round and 4 elongated tools), and the rest of the 8 animals and 8 tools in the HSF condition. During each run, there was a total of 32 mini-blocks. Each block showed eight exemplars of the same item (e.g., eight different squirrels) for 300 ms each, followed by a fixation for 615 ms. Participants performed a one-back task to detect immediate image repetition by pressing a response button with their right index finger. There was a single repetition that appeared randomly in each mini-block. A long fixation (7.32 s) was presented at the beginning of each run and after every four mini-blocks. Each mini-block lasted 7.32 s, and each run lasted 5 min. There was a total of eight runs. The experiment was run using Psychtoolbox in MATLAB (Brainard, 1997; Kleiner et al., 2007). The presentation order of the mini-blocks was randomized across runs and participants.

The localizer consisted of a single run of intact or scrambled animal or tool images in different blocks. There was a total of 32 blocks, with each block showing 10 images of the same category (animals/tools) and format (intact/phase scrambled) for 300 ms each, followed by a fixation for 615 ms. A 9.15 s fixation interval was presented at the beginning of each run and after every four blocks. A Latin-square was used to balance the block orders across participants. Participants performed a one-back task, where a single repetition appeared randomly in each block. The localizer lasted 6.25 min.

##### Imaging parameters.

The functional and anatomic MRI data were acquired on a Siemens Prisma 3T scanner equipped with a 64-channel head coil at the Center for Brain Imaging, New York University. The acquisition parameters of the echoplanar imaging T2*-weighted images were as follows: repetition time, 915 ms; echo time, 37 ms; flip angle, 52°; field of view (FoV), 208 mm (with FoV phase of 100%). Using the Center for Magnetic Resonance Research multiband sequence (Auerbach et al., 2013; Xu et al., 2013) with full-brain coverage and an acceleration factor of 8, each volume contained 72 slices, with 2 mm thickness and no gap. The image acquisition was tilted 30° clockwise from the anterior commisure-posterior comissure plane (Deichmann et al., 2003). An anterior to posterior phase encoding direction was used. Spin echo field maps and single-band reference image for each functional run were also collected before each functional run. Anatomical T1-weighted and T2-weighted 3D images were also collected. Each of the volumes contained 208 slices with 0.8 × 0.8 × 0.8 mm resolution, a matrix size of 320 × 320 (93.8%), and FoV of 256 mm.

##### Imaging data preprocessing and analysis.

Preprocessing of the imaging data followed the minimal preprocessing pipelines of the Human Connectome Project (Glasser et al., 2013). The pipelines included removal of spatial artifacts and distortions in anatomic and functional images, segmentation, precise within-subject registration between functional and anatomic images, cross-subject registration to the standard MNI volume space, and motion correction. Statistical Parametric Mapping (SPM12; http://www.fil.ion.ucl.ac.uk/spm) was then used for subsequent analyses. General linear model was used to estimate the blood oxygenation level-dependent (BOLD) response in the functional data, with each condition as the regressor of interest (32 in the main experiment and 4 in the localizer) and the 12 head movement parameters as the regressors of no interest. Spatial smoothing with a 4 mm FWHM Gaussian kernel was applied for univariate, but not for multivariate, analyses. For univariate analysis in the main experiment, the neural responses were averaged across the four items within each condition(e.g., LSF round animals). For data visualization, BrainNet Viewer (Xia et al., 2013) was used.

#### Definitions of regions of interest

**Figure 3.**
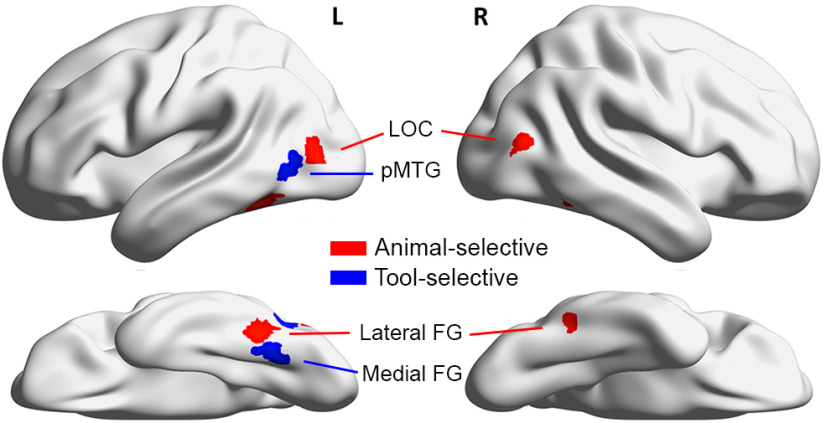
Category-selective ROIs of a representative participant. The ROIs were defined within each participant using an independent localizer in which animals and tools with varied shapes were shown to participants. Animal-selective ROIs included left and right LOC, and left and right lateral FG; tool-selective ROIs included left medial FG, and left pMTG. L, left hemisphere; R, right hemisphere.

The main focus of the analyses was in the category-selective regions of interest (ROIs). Using the localizer data, animal- and tool-selective ROIs were defined in each individual in the occipitotemporal cortex. Figure 3 illustrates the four animal-selective and two tool-selective ROIs of a representative participant. With the contrast of intact animals versus tools, animal-selective ROIs in bilateral lateral occipital complex (LOC) and bilateral lateral fusiform gyrus (FG), and tool-selective ROIs in the left medial FG and left posterior middle temporal gyrus (pMTG) were defined. All ROIs were defined with a threshold at *p* = 0.01 (uncorrected). If no clusters were found at this threshold, the threshold was lowered to *p* < 0.05 (uncorrected). For each ROI, the more lenient threshold was used for four or fewer participants. After locating the voxel with the peak activation in each ROI, a 10 mm radius sphere was drawn around the peak voxel. To ensure selectivity, only up to 75 voxels that showed the most significant effects with at least the threshold of *p* < 0.05 (uncorrected) were selected within each sphere. If <30 voxels reached the selection criterion of *p* < 0.05 (uncorrected), the data of the participant were excluded from the analyses of the particular ROI. The ROIs were successfully defined in a majority of participants (15–19 of 20). For the ROIs, the average number of selected voxels was 68 (left LOC: mean = 71 voxels, SD = 9.4; right LOC: mean = 70 voxels, SD = 11.6; left lateral FG: mean = 66 voxels, SD = 13.7; right lateral FG: mean = 73 voxels, SD = 9.2; left medial FG: mean = 57 voxels, SD = 17.5; left pMTG: mean = 73 voxels, SD = 6).

#### Univariate and multivoxel pattern analyses

Univariate analyses were conducted on the data within the ROIs. Random-effects whole-brain analyses were also performed to acquire group statistical maps for the main effects of Category, Shape, and Spatial Frequency, respectively.

Multivariate pattern analysis (MVPA) was performed in the ROIs in the following two ways: classification analysis with SVM using LibSVM software (Chang and Lin, 2011); and RSA with a toolbox by Nili et al. (2014).

For the classification analysis, a leave-one-out cross-validation procedure was used to decode neural response patterns among category (animals vs tools), shape (round vs elongated), and spatial frequency (low vs high) within each participant. One-sample *t* tests were then conducted on the mean accuracy across participants at the group level, against the 50% chance level. The pairwise comparisons of classification accuracy among the three factors were also conducted.

**Figure 4.**
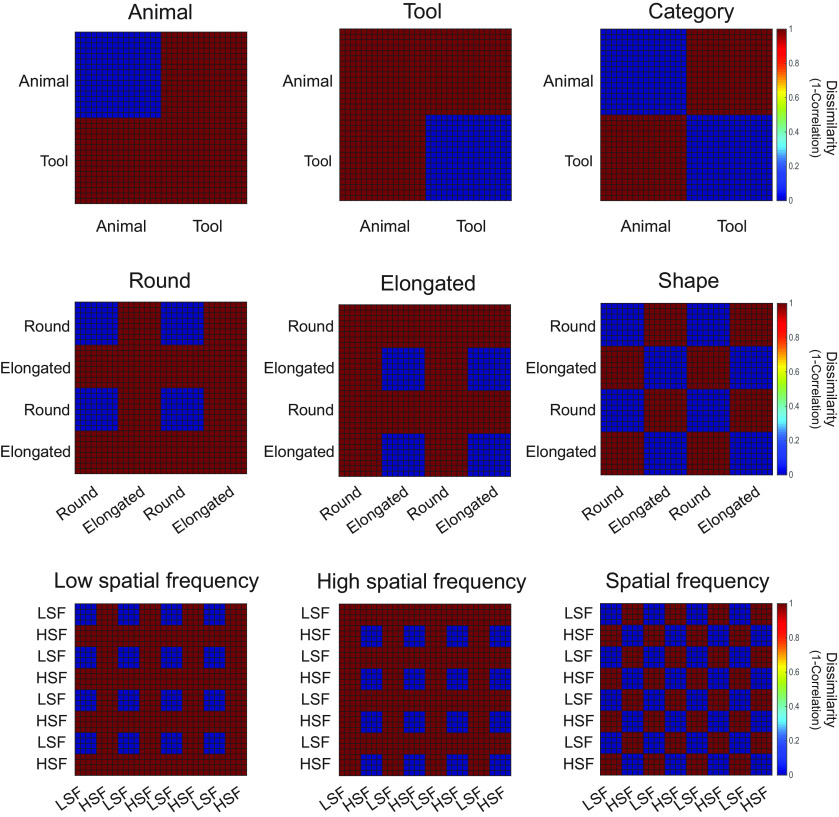
The nine hypothetical dissimilarity matrices (models) for items across the Category, Shape, and Spatial Frequency conditions. For each participant, a total of four items was randomly selected from a pool of 16 animals or 16 tools for each of the eight conditions in the experiment.

For RSA, t-maps of activations calculated for each item compared with the fixation baseline were used to form a 32 × 32 neural representational dissimilarity matrix (RDM) for each participant in each ROI, with the dissimilarity computed by 1 minus Pearson's *r* for each pairwise comparison across items. These RDMs were then compared with a total of nine models: Category, Animal, Tool, Shape, Round, Elongated, SF, LSF, and HSF, as illustrated in Figure 4. For the Category model, the dissimilarity was low among items of the same categories (e.g., within animals or within tools) but high for items of different categories (e.g., animals vs tools). For the Animal (or Tool) models, the dissimilarity was low only among items in that category, but the dissimilarity was high for items among the other category, or for items between the categories. The other six models (Shape, Round, Elongated, SF, LSF, and HSF) were constructed in a similar manner.

Within each ROI, the RDM of each participant was compared with each hypothetical model using Spearman's correlation with the RSA toolbox (Nili et al., 2014). The noise ceiling was also estimated for each ROI to assess the quality of the data (Nili et al., 2014). A one-sided Wilcoxon signed-rank test was then used to test the significance of each model at the group level by comparing the correlation values against 0 across participants. To compare the correlation results between different models, two-sided Wilcoxon signed-rank tests were used.

Whole-brain searchlight analysis using RSA was also performed to evaluate the correlations between the neural response patterns and the nine models, with a 6-mm-radius sphere generated for each voxel within the whole brain.

#### Follow-up study

We also conducted a follow-up study that was similar to the current study. Using a rapid event-related design, the follow-up study replicated the main findings of the current study. Another group of 20 healthy, right-handed undergraduate students at New York University Abu Dhabi provided informed consent and completed six runs of the main experiment and two localizer runs. Instead of a one-back task, here participants performed a size judgment task (whether the real-world size of an item was larger or smaller than a typical shoebox) on each image.

In the main experiment, each run consisted of 128 stimulus trials each lasting 1830 ms (300 ms image presentation + 1530 ms blank screen) and 84 jitter trials each lasting 915 ms (fixation). Four trials were included for each of the 32 conditions (2 SFs × 2 shapes × 2 categories × 4 items), with different exemplars shown in each run. Each exemplar image was shown twice in the experiment. Each run of the localizer consisted of 120 stimulus trials (300 ms image presentation + 1530 ms blank screen) and 80 jitter trials (915 ms), with 60 animals and 60 tools shown in either LSF or HSF. Each item was presented once in each run.

The functional and anatomic MRI data were acquired on a Siemens Prisma 3 T scanner equipped with a 64-channel head coil at New York University Abu Dhabi. All other aspects of the materials, design, scanning parameters, and data preprocessing and analysis were identical to the original study.

## Results

### Behavioral results

Performance in the one-back task was very high (Table 1). Although the visibility of the LSF and HSF images were comparable in the pilot study, a 2 × 2 × 2 ANOVA conducted on accuracy and RTs in the one-back task in the scanner revealed a significant effect of Spatial Frequency (accuracy: *F*_(1,19)_ = 4.52, *p* = 0.047; RT: *F*_(1,19)_ = 25.26, *p* = 0.0001), with better and faster performance for LSF than HSF images. There was also a significant interaction between Spatial Frequency and Shape in both accuracy (*F*_(1,19)_ = 5.71, *p* = 0.028) and RT (*F*_(1,19)_ = 8.05, *p* = 0.01), with a larger effect of Spatial Frequency for elongated than round shapes. There were no other significant effects or interactions (*p* values > 0.14).

**Table 1.** Behavioral results (mean accuracy and reaction time) of the main experiment

			Mean accuracy	Mean response time (ms)
	Round	LSF	0.95 (0.02)	533 (57)
Animal		HSF	0.95 (0.03)	550 (55)
	Elongated	LSF	0.95 (0.02)	525 (41)
		HSF	0.94 (0.02)	562 (47)
	Round	LSF	0.95 (0.03)	522 (55)
Tool		HSF	0.95 (0.02)	548 (41)
	Elongated	LSF	0.95 (0.02)	532 (47)
		HSF	0.94 (0.02)	564 (38)

The SDs are in the parenthesis.

### Univariate results

#### ROI analysis

**Figure 5.**
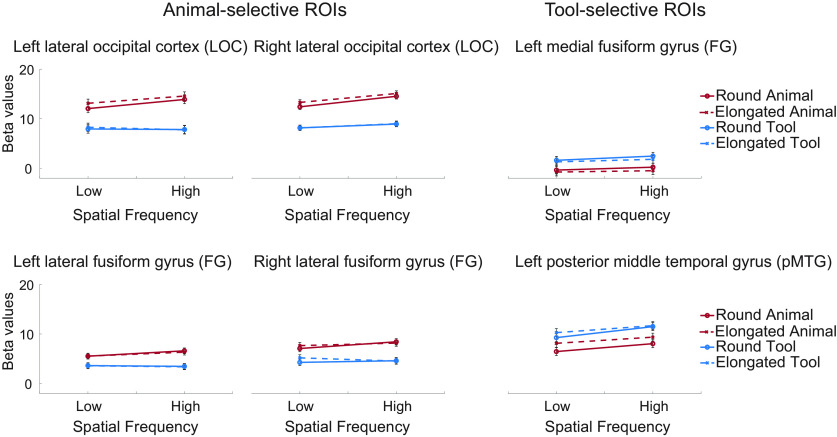
Univariate results in animal-selective and tool-selective ROIs. Averaged beta values of BOLD responses are shown for each of Category × Shape × Spatial Frequency conditions. Error bars indicate 95% confidence intervals of the three-way interaction.

To examine whether category selectivity is observed for images with comparable gist statistics across categories, and whether the category-selective regions also respond differentially for different shapes and spatial frequencies, a 2 × 2 × 2 ANOVA with the within-subjects factors Category (animals vs tools), Shape (round vs elongated), and SF (LSF vs HSF) was conducted on the amplitude of neural responses in each ROI. The results of all six ROIs are illustrated in Figure 5.

Robust category selectivity remained in all animal- and tool-selective ROIs. In all four animal-selective ROIs, animals evoked significantly stronger activations compared with tools (left lateral FG: *F*_(1,15)_ = 63.1, *p* < 0.0001; right lateral FG: *F*_(1,17)_ = 66.3, *p* < 0.0001; left LOC: *F*_(1,17)_ = 123.9, *p* < 0.0001; right LOC: *F*_(1,17)_ = 70.1, *p* < 0.0001). The interaction between SF and Category was also significant (left lateral FG: *F*_(1,15)_ = 6.6, *p* = 0.022; right lateral FG: *F*_(1,17)_ = 6.6, *p* = 0.02; left LOC: *F*_(1,17)_ = 7.9, *p* = 0.012; right LOC: *F*_(1,17)_ = 5.0, *p* = 0.039), with larger category differences for HSF than LSF images. In the right LOC, the mean response to HSF was significantly higher than that for LSF (*F*_(1,17)_ = 38.3, *p* < 0.0001). No other main effects or interactions were significant (all *p* values > 0.07).

In the two tool-selective ROIs, the activations were significantly stronger for tools than animals (left medial FG: *F*_(1,14)_ = 84.8, *p* < 0.0001; left pMTG: *F*_(1,18)_ = 45.3, *p* < 0.0001). The effect of Shape was also significant, with stronger activations for round than elongated shapes in the left medial FG(*F*_(1,14)_ = 5.7, *p* = 0.032), and stronger activations for elongated than round shapes in the left pMTG (*F*_(1,18)_ = 5.4, *p* = 0.032). In the left pMTG, the effect of Spatial Frequency was alsosignificant, with stronger activations for HSF than for LSF (*F*_(1,18)_ = 24.1, *p* = 0.0001). No other significant results were found (all *p* values > 0.09).

#### Whole-brain analysis

Random-effects group analyses in the whole brain (Fig. 6,Table 2) showed that Category, Shape, and SF largely activated separate brain regions (*q* < 0.05, FDR corrected for each contrast). Critically, the results confirmed the category-selective results in the ROI analysis, revealing stronger activations for animals than tools in bilateral LOC and lateral FG, and stronger activations for tools than animals in bilateral medial FG and the left pMTG. Additionally, animals also elicited stronger activations than tools in bilateral supramarginal gyrus, bilateral precentral, right middle frontal gyrus, right inferior parietal lobule, right amygdala, and bilateral precuneus. Stronger responses for tools than animals were found in the left middle occipital gyrus and left insula. Note that stronger responses for tools than animals were also observed in the bilateral superior parietal lobe (peak coordinates: left, −20, −62, 42; right, 26, −66, 42) and left inferior parietal lobe (peak coordinates: −46, −26, 46) at relatively lenient thresholds (*p* = 0.01 or *p* = 0.05 uncorrected, respectively).

**Table 2.** Results of random-effects group-level univariate analyses in the whole brain for the main effects of Category, Shape, and Spatial Frequency (*N* = 20, *q* < 0.05, FDR corrected)

	MNI coordinates			Number of voxels
Animals > tools	*x*	*y*	*z*
Left LOC	−52	−78	6	547
Right LOC	52	−70	4	695
Left lateral FG	−38	−40	−22	119
Right lateral FG	42	−42	−22	324
Left supramarginal gyrus	−58	−40	28	217
Right supramarginal gyrus	54	−30	34	1983
Left precentral	−36	−4	54	318
Right precentral	26	−4	60	460
Right middle frontal gyrus	56	8	44	61
Right inferior parietal lobule	34	−48	52	250
Right amygdala	20	−2	−16	18
Precuneus	6	−60	64	827
Tools > animals				
Left medial FG	−28	−58	−12	609
Right medial FG	26	−58	−12	569
Left pMTG	−52	−58	−8	25
Left middle occipital gyrus	−26	−84	10	115
Left insula	−34	−4	14	21
Round > elongated				
Left inferior occipital gyrus	−20	−92	−12	580
Right inferior occipital gyrus	24	−90	−8	293
Elongated > round				
Right middle temporal gyrus	50	−64	2	20
Left postcentral gyrus	−50	−28	40	20
LSF > HSF				
Left anterior temporal lobe	−62	−6	−14	30
Right anterior temporal lobe	54	0	−24	41
Right inferior orbitofrontal	50	32	−10	74
Bilateral medial frontal gyrus	−2	50	10	438
Left lingual gyrus	−12	−72	2	85
Right lingual gyrus	6	−60	0	42
Bilateral precuneus	−10	−72	24	1427
Left posterior cingulate	−6	−50	36	198
Right posterior cingulate	6	−52	26	84
Left inferior parietal lobule	−40	−70	46	247
Right inferior parietal lobule	50	−64	46	33
Left superior frontal gyrus	−24	48	30	59
Right superior frontal gyrus	14	54	38	70
HSF > LSF				
Left occipital lobe	−30	−88	−2	3063
Right occipital lobe	26	−94	−8	3205
Left superior occipital lobe	−26	−70	30	61
Right superior parietal lobe	30	−70	60	900
Left precentral gyrus	−48	2	34	59
Left inferior parietal lobe	−38	−44	46	247

**′Figure 6.**
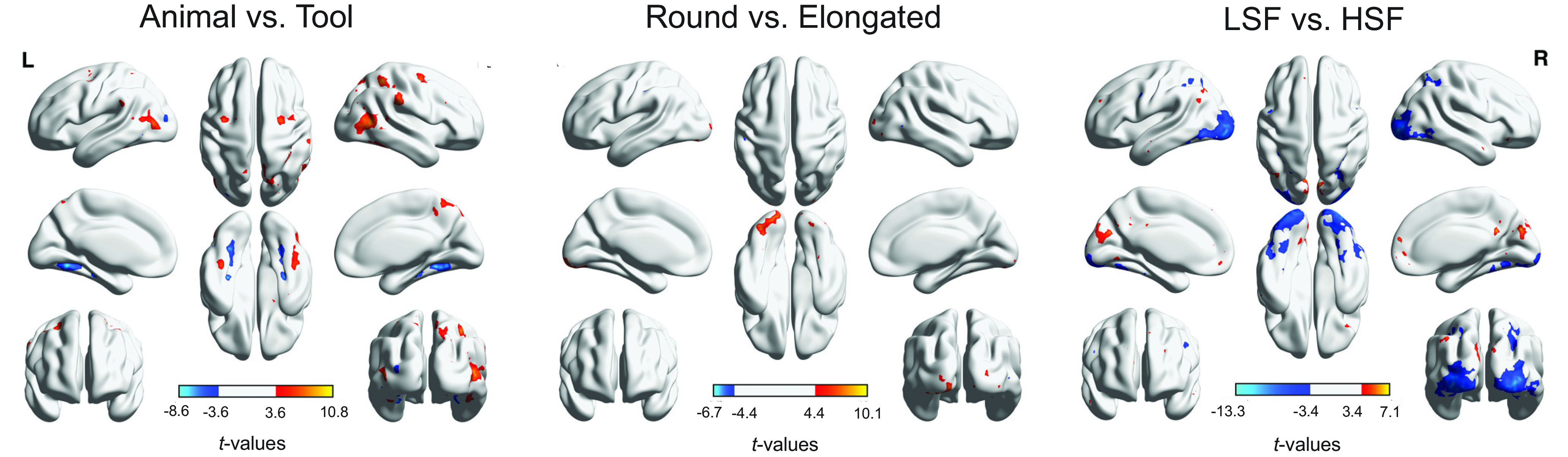
Whole-brain univariate results of the contrasts between animals and tools, between round and elongated shapes, and between low and high spatial frequencies in the main experiment (*q* < 0.05, FDR corrected for each contrast; warm colors: animals, round, LSF; cool colors: tools, elongated, HSF). L, left hemisphere; R, right hemisphere.

Although the effects of Shape and Spatial Frequency appeared less robust in the category-selective ROIs compared with that of Category, the whole-brain analysis revealed the effects of Shape and Spatial Frequency predominantly in the occipital region among others. Because we chose the stimuli primarily based on matching image statistics, one potential concern is that manipulations such as shape might lead to nonvisual differences between image sets that were correlated with visual shapes (e.g., action properties associated with round vs elongated tools). However, given that the neural differences due to shape arose mainly in the occipital cortex, this provides some evidence that our manipulation of shape was primarily tapping into visual information. Specifically, stronger activations for round than for elongated shapes were observed in bilateral occipital cortex and stronger activations for elongated than for round shapes were observed in small clusters in right middle temporal gyrus and left postcentral gyrus. Moreover, stronger activations for LSF than HSF images were observed primarily in bilateral medial frontal gyrus, bilateral precuneus, bilateral posterior cingulate gyrus, and bilateral inferior parietal lobule, whereas stronger activations for HSF than for LSF images were observed primarily in large portions of bilateral occipital lobe extending into inferior temporal cortex, right superior parietal lobe, and left precentral gyrus.

### Multivariate pattern analysis

#### Classification performance for Category, Shape, and Spatial Frequency in the ROIs

**Figure 7.**
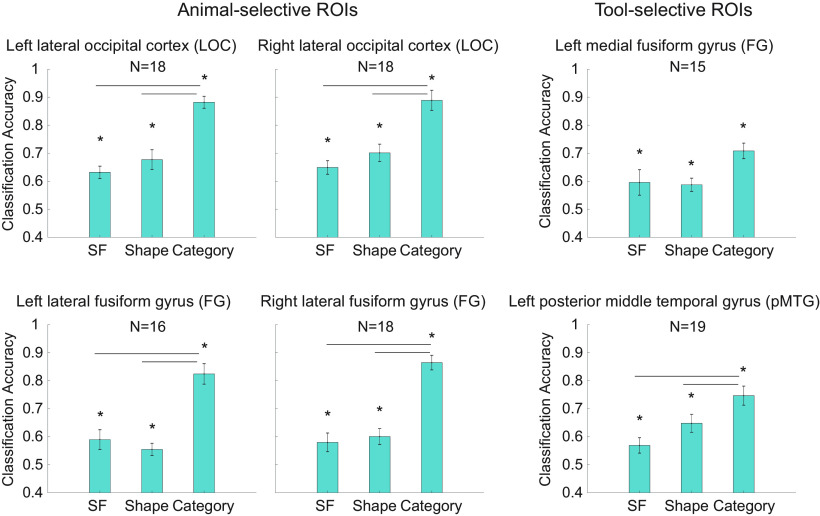
Averaged classification accuracy between Category (animals vs tools), Shape (round vs elongated), and Spatial Frequency (low vs high) in animal-selective and tool-selective ROIs. Error bars indicate SEM. Asterisks show significantly higher classification accuracy than the 50% chance level (*q* < 0.05, FDR corrected in each ROI). The black lines indicate significant pairwise comparisons among conditions, with higher classification accuracy for Category compared with Shape or Spatial Frequency in all animal-selective ROIs and in the tool-selective pMTG (*q* < 0.05, FDR corrected in each ROI).

We performed classification-based MVPA using SVM to further explore potential differences for Category, Shape, and Spatial Frequency in the ROIs, and confirmed that the effect of Category was the most robust among the three factors. The classification performance in the ROIs is illustrated in Figure 7. One-sample *t* tests conducted on the mean accuracy across participants compared with the 50% chance level revealed above-chance classification performance between categories (animals vs tools), shapes (round vs elongated), and spatial frequencies (LSF vs HSF) in all ROIs (*q* values < 0.05, FDR corrected in each ROI).

Moreover, one-way ANOVAs with the factor Condition (Category vs Shape vs Spatial Frequency) conducted on the classification accuracy revealed significant differences in all ROIs (left LOC: *F*_(2,34)_ = 28.8, *p* < 0.0001; right LOC: *F*_(2,34)_ = 18.1, *p* < 0.0001; left lateral FG: *F*_(2,30)_ = 31.1, *p* < 0.0001; right lateral FG: *F*_(2,34)_ = 49.0, *p* < 0.0001; left medial FG: *F*_(2,28)_ = 4.5, *p* = 0.02; left pMTG: *F*_(2,36)_ = 9.1, *p* = 0.0006). Subsequent pairwise comparisons revealed significantly higher accuracy for classifying category information, compared with shape and spatial frequency information in all animal-selective ROIs and in the tool-selective pMTG (*q* values < 0.05, FDR corrected). In the tool-selective left medial FG, the classification accuracy for Category was only numerically higher than that for Spatial Frequency (*p* = 0.47). In contrast, there was no statistical difference in classification performance between Shape and Spatial Frequency in any ROIs (*q* values > 0.1).

#### Representational similarity between neural response patterns and theoretical models in the ROIs

**Figure 8.**
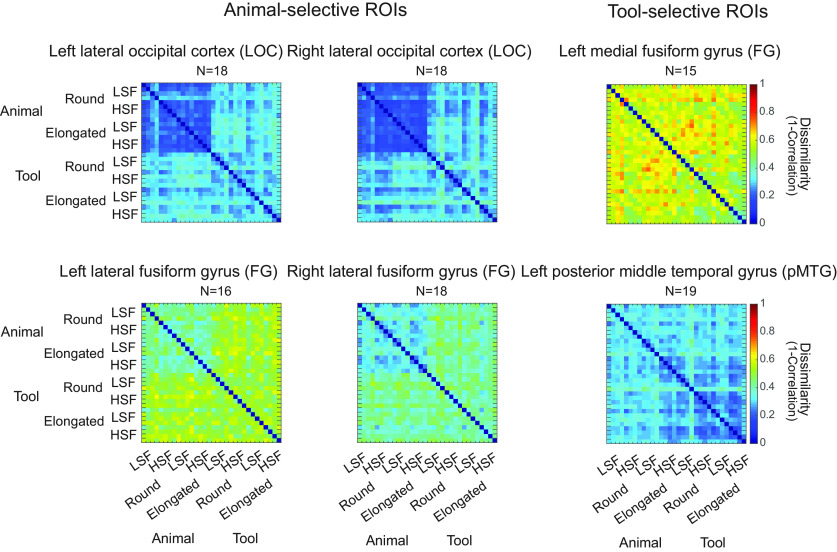
Each of the 32 × 32 matrices revealed averaged neural representational dissimilarity across Category, Shape, and Spatial Frequency, with four items included in each of the eight conditions, in the animal-selective and tool-selective ROIs.

As distinguishable neural response patterns were observed in the category-selective ROIs for animal and tool images that shared comparable gist statistics, we further asked how information about the categories is represented in these ROIs. Specifically, do animal-selective regions contain information of both animals and tools, or only of animals? We examined the nature of representation in the category-selective ROIs by computing the correlations between the neural response patterns (Fig. 8) and the theoretical models (Fig. 4).

**Figure 9.**
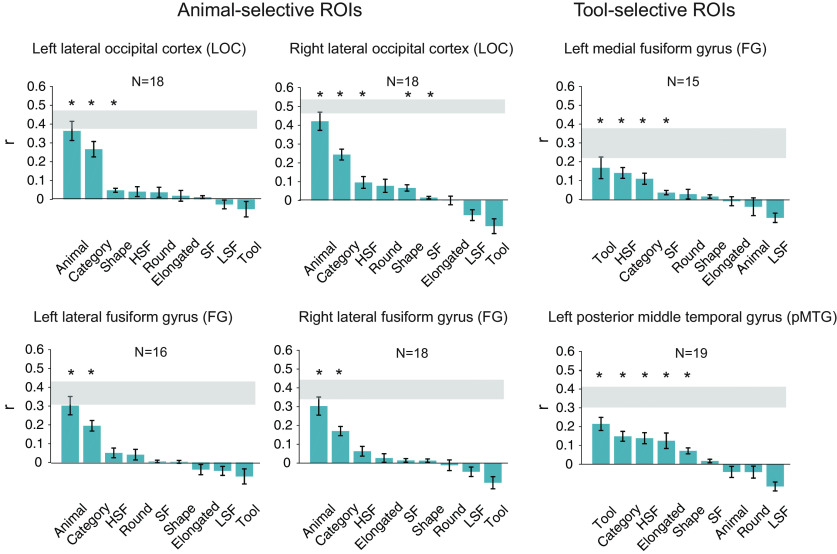
Correlations of the averaged neural representational dissimilarity matrices with the hypothetical dissimilarity matrices in the animal-selective and tool-selective ROIs in the main experiment. Asterisks indicate significant correlations (*q* < 0.05, FDR corrected); error bars indicate SEM. The gray bars represent the noise ceilings.

In all four animal-selective ROIs, the Animal model, which shows low dissimilarity among animals and high dissimilarity among tools and across animals and tools, was most correlated with the neural response patterns (Fig. 9; *r* values = 0.30–0.42, *p* values < 0.0001). In contrast, the neural response patterns in the two tool-selective ROIs were strongly correlated with the Tool model, which shows low dissimilarity among tools and highdissimilarity among animals and across animals and tools (*r* values = 0.17–0.21, *p* values < 0.0027). The Category model, which shows low dissimilarity among items within categories andhigh dissimilarity among items across categories, was also significantly correlated with the neural patterns in all six ROIs(*q* values < 0.05, FDR corrected). On the other hand, the correlations among the neural response patterns and other models were less consistent across the ROIs with the same statistical threshold (*q* < 0.05, FDR corrected; Fig. 9).

**Figure 10.**
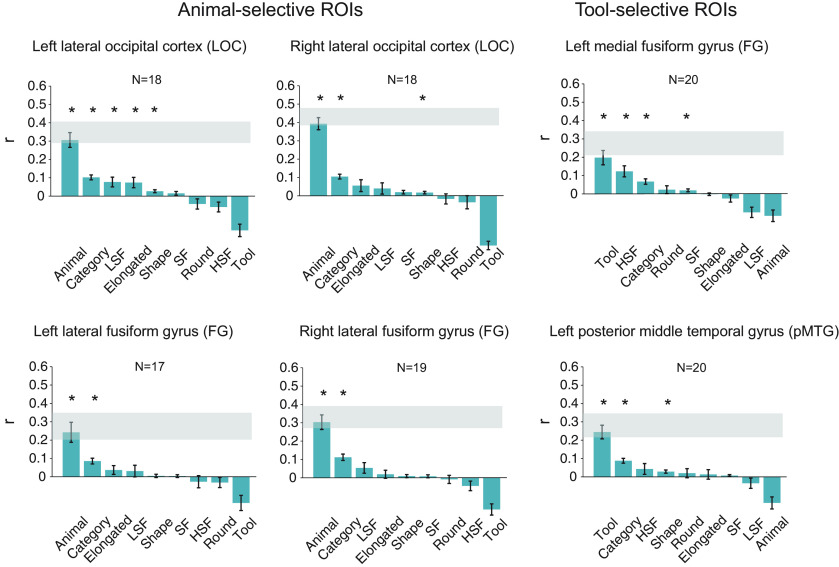
Correlations of the averaged neural representational dissimilarity matrices with the hypothetical dissimilarity matrices in the animal-selective and tool-selective ROIs in the follow-up experiment. Asterisks indicate significant correlations (*q* < 0.05, FDR corrected); error bars indicate SEM. The gray bars represent the noise ceilings.

We found that the Animal and Tool models were most robust in their respective selective ROIs. The correlations of the Animal model and the neural response patterns in the animal-selective ROIs approached or reached the corresponding lower bound of noise ceilings, and were significantly higher than all other models (*q* values < 0.05, FDR corrected for all comparisons in all ROIs except for the Animal model vs Category model in left LOC: *p* = 0.03, uncorrected). The Tool model significantly outperformed several models in both tool-selective ROIs (*q* values < 0.05, FDR corrected), although it was only numerically more correlated with the neural response patterns compared with the HSF and Elongated models in left pMTG, and compared with the Category, HSF, SF, Round and Animal models in the left medial FG. Nonetheless, in the follow-up event-related study (Fig. 10), significant correlations between the Tool model and the neural response patterns in both tool-selective ROIs were also observed (*r* values > 0.2, *p* values < 0.0001), while the significant correlations between the Animal model and the neural response patterns were also found in all four animal-selective ROIs. Critically, the correlations of the Animal model or the Tool model with the neural response patterns were significantly higher than the correlations for all other models, including the Category model, and the neural response patterns in their respective ROIs (*q* values < 0.05, FDR corrected).

#### Whole-brain searchlight analysis using RSA

**Figure 11.**
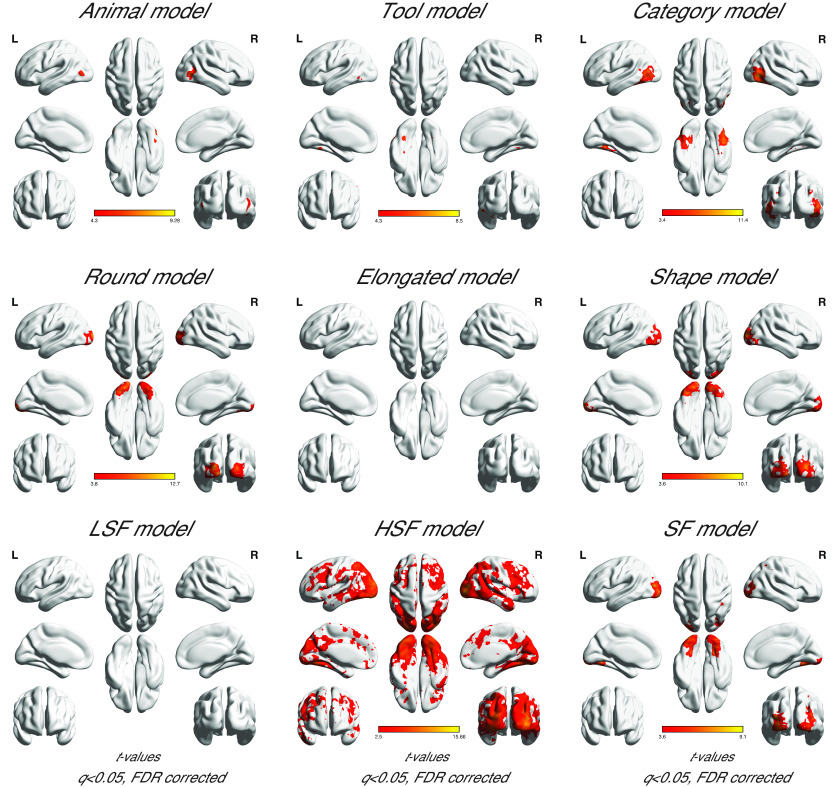
Whole-brain searchlight results for significant correlations between neural pattern responses and the Animal, Tool, Category, Round, Elongated, Shape, LSF, HSF, and SF models, respectively, in the main experiment (*q* < 0.05, FDR corrected within each model). Critically, the significant clusters for the Animal and Tool models were found within the clusters for the Category model. L, left hemisphere; R, right hemisphere.

The whole-brain searchlight analysis was performed (Fig. 11, Table 3) to examine whether the animal- and tool-selective responses may be limited to clusters around the regions with peak selectivity in the occipitotemporal cortex, or also more generally in other regions. With a threshold of FDR-corrected *q* < 0.05 performed with the Animal and Tool models separately, we again found that animal information was represented within bilateral LOC, bilateral lateral FG, and additionally in the right precentral region, while tool information was again represented in bilateral medial FG, left pMTG, and additionally left inferior parietal lobule and left superior parietal lobule. Interestingly, the Category model was found to be significant in bilateral occipitotemporal cortex, which showed larger clusters than the occipitotemporal clusters found for the Animal or Tool models, and also in right inferior and middle frontal gyrus, right precuneus, bilateral inferior parietal lobule, right precentral, right supramarginal gyrus, and right angular gyrus.

**Table 3.** Whole-brain searchlight results for the Animal, Tool, Category, LSF, HSF, SF, Round, Elongated, and Shape models (*N* = 20, *q* < 0.05, FDR corrected within each model)

	MNI coordinates			Number of voxels
Animal model	*x*	*y*	*z*	
Left lateral occipital complex	−54	−72	4	328
Right lateral occipital complex	54	−70	2	1590
Left lateral fusiform gyrus	−38	−40	−22	36
Right lateral fusiform gyrus	50	−26	−22	22
Right precentral	38	0	40	37
Tool model				
Left medial FG	−26	−58	−14	314
Right medial FG	28	−50	−10	80
Left pMTG	−52	−58	−6	101
Left inferior parietal lobule	−58	−30	44	32
Left superior parietal lobule	−26	−62	42	25
Category model				
Left occipitotemporal cortex	−38	−58	−10	6014
Right occipitotemporal cortex	42	−64	−10	5145
Right inferior frontal gyrus	50	34	12	22
Right middle frontal gyrus	44	18	26	37
Right precuneus	28	−52	52	35
Right supramarginal gyrus	58	−22	32	33
Right angular gyrus	30	−58	48	39
Right precentral	38	−2	54	33
Left inferior parietal lobule	−40	−36	46	34
Right inferior parietal lobule	38	−38	48	101
LSF model				
None				
HSF model				
Occipital-temporal-parietal-frontal cortex	36	−80	14	86467
Bilateral rectus	6	20	−24	55
Left middle frontal gyrus	−32	60	−10	62
Right caudate	14	−2	16	264
Right thalamus	6	−20	8	184
Bilateral paracentral lobule	−2	−38	76	66
SF model				
Left occipital lobe	−34	−96	10	3291
Right occipital lobe	42	−78	10	3305
Right inferior parietal lobule	40	−40	46	89
Right superior parietal lobule	30	−54	54	94
Round model				
Left occipital lobe	−20	−96	8	2195
Right occipital lobe	24	−88	−8	2216
Elongated model				
None				
Shape model				
Bilateral occipital lobe	28	−90	−8	8016
Left postcentral	−56	−30	54	21
Precuneus	0	−74	52	67
Right superior parietal lobule	24	−58	54	50

**Figure 12.**
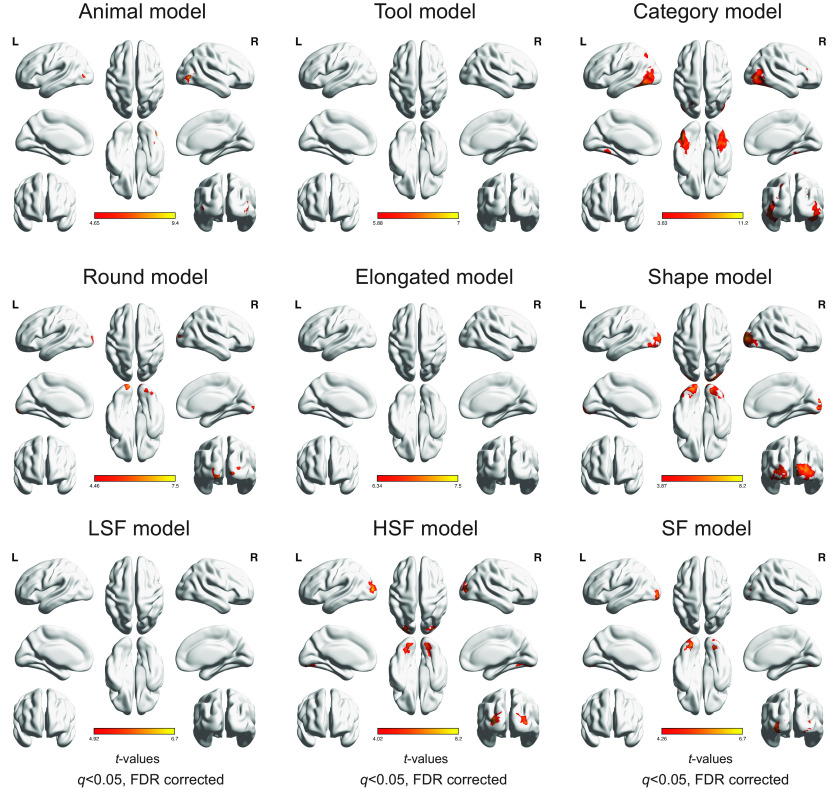
Whole brain searchlight results for significant correlations between neural pattern responses and the Animal, Tool, Category, Round, Elongated, Shape, LSF, HSF, and SF models, respectively, in the follow-up study (*q* < 0.05, FDR corrected within each model). L, left hemisphere; R, right hemisphere.

Additional analyses performed to examine the representations of shape and spatial frequency information revealed that such information was represented in regions more posterior than the category-selective ROIs. The Shape model was significantly correlated with clusters in bilateral occipital cortex, precuneus, left postcentral gyrus and right superior parietal lobule. The Round shape model showed correlations with clusters in bilateral occipital lobe, while no significant clusters were found to be significantly correlated with the Elongated shape model. The Spatial Frequency model was correlated with clusters in bilateral occipital lobe extending into the medial part of inferior temporal cortex, and right inferior and superior parietal lobule. The correlation with the LSF model did not reveal any cluster surviving FDR correction, while representation of HSF information was found in extensive brain regions spanning occipital-temporal-parietal-frontal lobes, as well as bilateral rectus, left middle frontal gyrus, right caudate, right thalamus and bilateral paracentral lobule. Such extensive responses for HSF information was unexpected—one possible explanation is that the extensive significant correlations with the HSF model might be related to the slightly worse behavioral performance for HSF than LSF images in the one-back task. Although this result might raise potential concerns on the comparability of the conditions, it is important to note that the behavioral performance was very high for both HSF and LSF images in the one-back task, and this result of the HSF model was not found in the follow-up study, where no behavioral difference between LSF and HSF images in the size judgment task was observed. Instead, the HSF model was only found to be correlated with clusters in bilateral occipital cortex (Fig. 12). More importantly, these results could not account for the main findings of the animal- and tool-selective effects, or the general trend of posterior-to-anterior progression of shape and spatial frequency information to category information in the occipitotemporal cortex.

#### Additional comparison of univariate and classification results in the ROIs between the original study and the follow-up study

Apart from the RSA results of the follow-up study, the findings for univariate and SVM analyses were also generally consistent between the original study and the follow-up study, particularly in terms of the effects of Category. Briefly, the SVM classification analysis showed highly comparable results between the two studies. For the univariate analysis, when comparing across the two studies with four-way ANOVAs with the factors Experiment (original vs follow-up), Category (animals vs tools), Shape (round vs elongated), and SF (LSF vs HSF), the overall responses and the category effects were stronger for the follow-up study in several ROIs, with significant main effects of Experiment in animal-selective bilateral FG, and tool-selective left medial FG (*F* values > 9.5, *p* values < 0.005) or significant interactions of Experiment and Category in animal-selective right LOC, and both tool-selective ROIs (*F* values > 4.6, *p* values < 0.05). The interaction between Experiment and SF was significant in the animal-selective ROIs (*F* values > 4.37, *p* values ≤ 0.04; except for left fusiform gyrus, *F*_(1,31)_ = 3.67, *p* = 0.065), revealing a trend of higher amplitude for HSF than LSF stimuli in the original study, but the opposite trend in the follow-up study. There was also an interaction between Experiment and Shape in animal-selective right LOC (*F*_(1,34)_ = 3.65, *p* = 0.023), with stronger responses for elongated than for round items in the follow-up study (*p* = 0.0002) but not in the original study (*p* = 0.43). Last, the interaction of Experiment, Shape, and Category in three animal-selective ROIs (bilateral lateral FG and right LOC: *F* values > 4.13, *p* values < 0.05) revealed comparable category effects for round and elongated items in the original study (*p* values > 0.22) whereas category effects appeared to be stronger for elongated than for round items in the follow-up study (*p* values ≤ 0.01; except for right lateral FG: *p* = 0.058). Note that none of the differences in the univariate results between the original study and the follow-up study affect our main conclusions, including the consistent category-selective effects, as revealed in the univariate analyses, the generally stronger effects of category than shape or SF, as revealed in the SVM analyses, and the specific representation of category information in the respective ROIs, as revealed in the RSA.

## Discussion

To examine the nature of category selectivity for animals and tools, we manipulated category, shape, and spatial frequency, and used images of animals and tools with comparable gist statistics across the categories. We found that the category effects in the animal- and tool-selective ROIs were independent of low- and mid-level visual features such as gist statistics, shapes, spatial frequencies that often covary with animals or tools, and that the representational content in the category-selective ROIs reflect information about the preferred category only, whereas the larger regions surrounding the ROIs showed sensitivity to both the preferred and nonpreferred categories.

### Distinguishing influences of category, shape, and spatial frequency

While gist statistics predict category-selective responses in the occipitotemporal cortex (Rice et al., 2014; Watson et al., 2014; Coggan et al., 2016, 2019), our univariate and multivariate analysis results show that minimizing differences in gist statistics that covary with category membership do not eliminate category-selective effects. Specifically, in the ROIs that were defined separately using images of animals and tools with naturally varied gist statistics, univariate analyses conducted in the ROIs revealed stronger activations for animals than tools in the animal-selective bilateral LOC and lateral fusiform gyri, and stronger activations for tools than animals in the tool-selective left pMTG and medial fusiform gyrus for images of animals and tools with comparable gist statistics. Whole-brain univariate analysis also revealed significant magnitude differences between animals and tools in lateral occipital complex and fusiform gyrus but not in the early visual cortex, further suggesting that the category effects observed here were not due to low- or mid-level visual differences between the categories. In contrast, univariate analyses revealed significant differences between round and elongated shapes, and between LSF and HSF in early visual cortex.

While the univariate effects for Shape and Spatial Frequency were not as reliable and consistent as the category effects in the ROIs, classification analyses (SVM) revealed that the neural response patterns were distinguishable not only between categories, but also between shapes and between SFs in most ROIs. These results are consistent with previous findings that both visual and category differences contribute to the neural responses in the occipitotemporal cortex (shape: Op de Beeck et al., 2008; spatial frequency: Vaziri-Pashkam et al., 2019; category: Haxby et al., 2001; Cox and Savoy, 2003; Pietrini et al., 2004; Kriegeskorte et al., 2008), and that the contributions of visual and category information may be independent (Bracci and Op de Beeck, 2016; Proklova et al., 2016). Extending from these findings, the current study directly compared the relative contributions of these factors to the representations in the animal- and tool-selective ROIs. The consistently higher classification accuracy for Category compared with Shape and Spatial Frequency in all ROIs suggests that the representations in these ROIs primarily reflect category information.

We used RSA to further reveal that the representational content in the ROIs was primarily driven by the preferred category of the ROIs. Specifically, the neural response patterns were correlated among different animals in the animal-selective ROIs and the neural response patterns were correlated among different tools in the tool-selective ROIs, whereas different items from the nonpreferred category were not correlated with each other. Moreover, the correlations of the neural response patterns were consistently higher for the Animal model in the animal-selective ROIs and for the Tool model in the tool-selective ROIs in most cases, compared with the Category model. Furthermore, the performance of the Animal and Tool models either reached or approached the noise ceiling in the animal- and tool-selective ROIs, respectively. Together, these results suggest that the category-selective ROIs primarily reflect information of the preferred category only, and not necessarily both preferred and nonpreferred categories, nor visual features.

### Specialized versus distributed category representations

It is important to emphasize that the category effects in all the ROIs across the univariate, SVM, and RSA analyses were observed and replicated in both experiments. While previous findings suggest that LOC is sensitive to shapes and fusiform gyrus is sensitive to both shapes and semantic information (Grill-Spector et al., 2001; Simons et al., 2003; Grill-Spector and Malach, 2004), here we found similar representations in both bilateral LOC and lateral fusiform, with the Animal model significantly outperformed all other shape or spatial frequency models in animal-selective ROIs. These results are consistent with previous findings on the large-scale organization in the occipitotemporal cortex with regard to animacy for LOC and lateral fusiform (Konkle and Caramazza, 2013), and suggest that while influences of visual information are represented in much of the occipitotemporal cortex (Bracci et al., 2019), the representational content within these ROIs is primarily based on animal information. Similarly, while the tool-selective left medial fusiform and left pMTG also showed significant effects between round versus elongated shapes and between LSF and HSF, consistent with previous findings (Mahon et al., 2013; Fabbri et al., 2016; Chen et al., 2018), our findings revealed that the most predominant representations in the tool-selective ROIs appear to be tool information.

While the notion of specialized representations for a preferred category (Kanwisher, 2010) is supported by the current results in the animal- and tool-selective ROIs, the RSA whole-brain searchlight analysis revealed significant correlations of the Category model with the neural response patterns in substantial areas in the occipitotemporal cortex, including and surrounding the category-selective ROIs (see also Clarke and Tyler, 2014). Since the Category model assumes that both animal and tool information is represented, these results appear to be consistent with the view of distributed representations for the categories in bilateral occipitotemporal cortex (Haxby et al., 2001). Future studies may further examine the functional organization of the focal category-selective areas being part of a larger cortical region with distributed category representations in the occipitotemporal cortex, and how the category-selective regions and the larger surrounding regions may interact to represent information about various categories.

### Role of higher-level processes in category selectivity

Since category representations in the animal- and tool-selective regions do not necessarily depend on low- and mid-level visual features including spatial frequencies, shapes, and gist statistics, what aspects of the preferred categories are represented in these ROIs? It is possible that higher-level cognitive processes, such as interpretation or categorization of the visual inputs modulate neural activations in the occipitotemporal cortex. For instance, the fusiform “face” area is activated when the nonface images are misperceived or interpreted as faces (Cox et al., 2004; Op de Beeck et al., 2006; Summerfield et al., 2006; see also Bentin et al., 2002; Hadjikhan et al., 2009). While observers readily associate animate versus inanimate attributes to novel objects based on salient visual features commonly found in either animate or inanimate categories (e.g., symmetry, color, texture; Cheung and Gauthier, 2014), the interpretation of an identical set of stimuli as either animate or inanimate entities via context or short-term learning leads to selective activations in lateral versus medial fusiform regions (Martin and Weisberg, 2003; Martin, 2007; Weisberg et al., 2007; Wheatley et al., 2007; see also James and Gauthier, 2003, 2004). Extending these findings, our results suggest that interpretations or categorization of visual inputs do not necessarily depend on low- or mid-level visual features.

Successful categorization of a visual input as an animate or inanimate entity may instead require knowledge about diagnostic features or feature combinations that are common to a particular category (e.g., ensemble of a head, a body, and four limbs for animals; Delorme et al., 2010). Indeed, the animal- and tool-selective ROIs appear to support categorical knowledge and not the relationship among the individual items. Specifically, the category-selective responses observed here were unlikely to be specific a limited set of visual features of particular items or exemplars, as a diverse image set of items and exemplars was used (Fig. 1; He and Cheung, 2019). Moreover, a follow-up analysis showed that the semantic associations among individual items calculated based on Latent Semantic Analysis (Landauer et al., 1998) yielded significantly lower correlations with the neural activations patterns than the Animal or Tool models in all the animal- and tool-selective ROIs, respectively (*q* values < 0.05, FDR corrected; *p* < 0.05 uncorrected in left medial fusiform). While a lateral-to-medial continuum in neural responses in the ventral pathway has been reported for animacy (Connolly et al., 2012; Sha et al., 2015), our RSA results showed high correlations in the neural response patterns among different animals or different tools within the ROIs. Together, these results suggest that categorical knowledge may be critical in shaping the selectivity. Future research may further elucidate the representational differences for categories versus individual members within the categories along the posterior-to-anterior axis along the ventral pathway (Clarke and Tyler, 2014).

### Conclusion

In sum, this study provides a new insight that the robust category selectivity in the occipitotemporal cortex does not solely depend on low-level or mid-level visual features that covary with the categories. Instead, the neural representations in the category-selective ROIs are likely based specifically on the general knowledge about a wide range of members of the preferred categories. We suggest that the category-selective regions may play a critical role in transforming visual inputs into conceptual knowledge.
